# Challenges and innovations in access to community‐based rural primary care services during the Covid‐19 pandemic in Australia

**DOI:** 10.1002/hpm.3598

**Published:** 2022-11-28

**Authors:** Belinda O’Sullivan

**Affiliations:** ^1^ Monash University School of Rural Health Bendigo Victoria Australia; ^2^ The University of Queensland Rural Clinical School Toowoomba Queensland Australia

**Keywords:** access, Covid‐19, doctor, General Practice, primary care, rural, telehealth

## Abstract

**Background:**

Access to primary care is a significant issue for rural populations. The Covid‐19 pandemic imposed a unique operating environment for rural General Practice enabling accessible services. This study aimed to explore the challenges and innovations rural General Practices experienced in promoting accessible primary care during a year of the pandemic.

**Methods:**

Longitudinal semi‐structured interviews were done with key informants (General Practitioners or Practice Managers) from purposefully selected General Practices from different rural towns in different subregions. Interviews occurred at three stages of the pandemic, June 2020–June 2021. They explored participant perspectives of the emerging challenges and innovatinos as they sought to support accessible primary care services during the pandemic. The data were thematically coded using a deductive framework of access challenges and innovations over time.

**Results:**

Of 12 practices approached, 11 key informants responded, providing around 30 h of interview data. The challenges and innovations related to access, changed over time as the pandemic evolved. A common theme concerned reflexive action. Practices had been on a journey during the pandemic to embed new planning processes, digital health options and to innovate to protect and support patients and staff to sustain access.

**Conclusion:**

This study provides useful insights into the challenges and innovations experienced in rural general practice during the Covid‐19 pandemic to reflect on models, strategies and approaches that can apply to promote access to rural primary care services going forward.

## Highlights

- Longitudinal interviews with key informants (General Practitioners and Practice Managers) from different types of rural General Practices in different rural towns, about the Covid‐19 response.
- Major access challenges include service pressures with limited staffing and changing patient needs over time.
- Reflexive action and planning helped practices to innovate and sustain access during pandemic challenges.

## 1 INTRODUCTION

Rural General Practices (practices), which consist of doctors and practice staff, play a critical role in delivering primary care services, protecting the most vulnerable people by providing local care to rural communities, overcoming the barriers of distance and cost.^1^ During the Covid‐19 pandemic, Australian General Practices, which work as independent business (Box 1) were faced with enormous pressure to evolve their services to ensure access to primary care, within a new operating environment. Specific to practices based in rural and regional settings, the pandemic issues came atop of existing pressures of operating with more limited workforce and promoting accessible services for clients with complex needs, who are spread over long distances.^2^, ^3^ Primary Health Networks (PHNs) were an important part of Australia's support system for General Practices at this time (Box 1). There are 31 PHNs across Australia who are independent agencies who work with General Practice and other stakeholders to strengthen and coordinate primary care services to deliver high quality primary care services which meet the needs of communities.^4^ This study was led by Murray PHN which has responsibility for a wide rural and regional catchment of 22 local government areas and around 200 General Practices spanning the north west to north eastern corners of Victoria (Box 1). It is located in the state hardest hit with the pandemic, Victoria (Box 1). The pandemic situation presented an opportunity for Murray PHN to explore the service challenges and innovations for practices related to maintaining access for rural communities. Aggregating the learning from doctors and Practice Managers who work in different types of practices in different subregions and exploring their perspectives over time was expected to inform the Murray PHN and its advocacy for wider system responses so as to meet the needs of rural General Practices and support accessible services for the community.

**Box 1: Summary of Australian primary care system and role of PHNs**

In Australia, the primary care sector operates mainly as a set of independent businesses which have the opportunity to set their own fees.

Patients have the opportunity to receive services at no cost if doctors choose to keep their fees at the level that a national universal health insurance scheme pays for services on a Medicare Services Schedule.^5^

Overall, 31 PHNs across Australia play a role in promoting General Practice services that are high quality and accessible to patients, particularly for at risk groups.^4^

The Murray PHN is based in regional Victoria, in the state which had the most significant rates of Covid‐19 infection and death Australia‐wide and.^6^ During the pandemic it continued to work with its 200 General Practices and other stakeholders to promote high quality, accessible primary care services for rural and regional communities by responding with information and resources.

Meanwhile, its catchment was subject to protracted lockdowns and border controls, new infection control protocols and immunisation requirements.

The regulations imposed on Murray PHNs' General Practices were controlled by the states under Australia's public health law, whereas the Commonwealth (federal government) were responsible for pandemic resources for General Practices including providing protective equipment and expanding Medicare to allow for telehealth billing.^7^, ^8^ The states co‐managed immunisations with the Commonwealth.

The Murray PHN had a strong interest in responding to access challenges and sponsored this study to learn more about the rural and regional practice experience and innovations that are possible to scale up.

The Commonwealth (federal) government is the main funder of PHNs and recognises that a coordinated response by General Practices as critical to the overall Covid‐19 response.^5^, ^9^ In rural settings General Practice is the main medical service that is available to people.^10^ Meanwhile, under law, the Victorian government sets public health regulations, such as state‐mandated lockdowns and co‐funds immunisation with the Commonwealth. Despite extensive policymaking and resourcing by state and federal government in response to different stages of the pandemic in Australia, there was limited information about the challenges and innovations in rural and regional General Practices and how they innovated to meet the primary care needs of local communities. Such information has the potential to inform how to strengthen rural and regional primary care which is a critical issue given that around half of the world's population^11^ and 30% of Australia's population that lives rurally.^12^

Several national‐scale Covid‐19 specific cross‐sectional surveys were done in Australia in 2020, describing patterns of response in General Practices. These suggested that practices rapidly pivoted from a normal business model in the early pandemic, including introducing a range of innovations in infection control, telehealth and additional prevention services to meet the needs of practice staff and patients.^6^ The results also suggested that rural communities encountered different challenges than those in metropolitan areas, such as issues with managing patient transfers.^6^ Metropolitan practices by comparison, were more likely roster more staff in response to the pandemic pressures, do less ambulatory services and ask colleagues for resources.^6^ With regard to sourcing more staff during the pandemic, other research suggests that rural practices have ongoing workforce recruitment and retention challenges which increase by rurality.^13^, ^14^ Rostering more staff may not have been a viable option in rural areas, including when international border closures which Australia imposed in 2020 may have affected the capacity to access overseas‐trained doctors on which many rural areas rely.^15^ Under normal conditions, rural doctors are also known to work in more isolated and busy conditions (longer hours), particularly when managing both practice and on‐call and hospital specialist rosters in an environment where there are limited other doctors.^16^, ^17^, ^18^ Perhaps highlighting the busy‐ness of General Practice in the rural and regional context, a further national cross‐sectional survey during the pandemic identified that these practices may have been less likely than those in metropolitan areas to experience a fall in patient numbers and a loss of income during the pandemic.^19^

An international perspective equally noted that rural and regional General Practices may have nuanced pandemic risks, resilience and need context‐based responses that need to be accommodated as part of pandemic planning.^10^ These include managing pandemics atop of regular care for a population with poorer health and socio‐economic status, longer distances, different population interaction patterns, reliance on mobile workforces^15^ and limited local technical infrastructure.^10^ As such the integration of any pandemic‐related health service requirements into rural systems is likely to have required specific framing, expertise and adjustments to meet the needs of the practice and the community.^20^ This fits with other research, showing that outside of pandemic situations, rural primary care needs to respond to constant adaptive pressures to address risk and capitalise on opportunities which help them to remain viable in the face of a changing health system environment.^21^ Understanding the level of challenges and innovations in rural and regional General Practices during the course of the Covid‐19 pandemic, could provide rich information for rural‐sensitive national and state pandemic policy and planning.

Finally, there have been a range of published narratives which are enthusiastic about learning from the pandemic to re‐set a ‘new normal’^22^, ^23^ but it is important to understand the layers and context of the ‘new normal’ with regard to rural General Practices using empirical data, before any widespread change, is espoused. To understand this better it is relevant to use longitudinal research methods which are powerful for exploring reactive and adoptive processes within complex and dynamic health systems.^24^

With this background in mind, this study aimed to explore the challenges and innovations of rural General Practices in promoting accessible primary care during 1 year of the pandemic in a state that was hardest hit with Covid‐19.

## 2 METHODS

### 2.1 Context

The study was funded by the Murray PHN (Box 1).^4^

### 2.2 Procedure and semi‐structured interviews

The study had ethics approval from Monash University, Project ID: 24918. It involved semi‐structured interviews over an annual period 2020–2021, including three different stages of the pandemic's evolution (Table 1). Longitudinal interviews allow for the exploration of unpredictable phenomenon which change over time as they are identified, tested, refined and embedded under real‐life conditions.^24^

**TABLE 1:** Pandemic timeline underpinning this study

Stage of the pandemic	Conditions
Stage 1—June 2020	State‐wide lockdown (stay at home orders), work from home orders, with protocols around managing Covid‐19 clinical risk including use of screening and personal protective equipment and using telehealth
	GPs pivoted to telehealth models mainly due to infection risk (initially with no funding for this).
	New government‐funded telehealth item number eventually introduced for GPs; practices and required practices to bulk bill (not change out of pocket costs) for it (fee set)^25^
Stage 2—December 2020	State‐wide lockdown (stay at home orders) eased, but workplaces and venues still running at capacity limits to prevent spread of Covid‐19
	Population had moved to regions as many able to work from home, resulting in rural population growth
	Telehealth item expanded (more eligibility, phone and video) and practices permitted to charge patients out of pocket fees for it^25^
Stage 3—June 2021	Vaccination roll‐out commenced, and practices enroled to supply vaccinations through an expression of interest process to the Government (gained accreditation to deliver)
	Vaccination supply limited to 50 vaccines per practice per week initially due to national supply chain limitations, then expanded based on practice utilisation data
	Guidelines for safety of various vaccines evolving within national policy (perceived risks creating vaccine hesitancy by community)
	Vaccination programme implemented in staggered way based on infection risk in pre‐set phase
	Telehealth item available ongoing, but limited to phone (not including video)

The study was advertised through the Murray PHN newsletter provided to all 200 General Practices in the region to promote communication with Practices who were very busy at the time of the pandemic. A purposeful sample of 12 different types of Practices from different subregions was then directly approached and asked to nominate a key informant, who was a General Practitioner or Practice Manager. The purposeful sampling aimed to broaden the range of perspectives of access issues by different contexts (rurality, practice size, models of care or managing specific populations and subregions). Participants read the explanatory statement and completed written consent for three rounds of interviews.

The interviews were up to an hours' duration, based on the participant conveying they had nothing more to add. Given the purposeful sampling, diversity of practices and changing pandemic context, saturation of the data may not have been achieved. Interviews were conducted by phone by an experienced rural health researcher who was contracted by the Murray PHN to conduct the research. The researcher did not know the participants and had no pre‐conceived ideas about what findings might emerge given the lack of published literature about rural General Practice experiences in the Covid‐19 context. Participants received a $100 gift card for each round of interviews they completed. Follow‐up interviews occurred by the Murray PHN re‐notifying the participants that the researcher would contact them.

The interview guide was developed by the researcher collaborating with the Murray PHN staff so as to optimally inform Murray PHN policies as well as the broader literature. The guide was revisited between each round of interviews to update the questions so as to expand on the findings of previous rounds of interviews and to understand emerging phenomenon as the pandemic situation changed (Table 2). Participants were asked to describe their practice's current services and community, models of care they used, workforce, challenges and innovations related to addressing the needs of various sub‐populations and sustaining care under evolving pandemic conditions.

**TABLE 2:** The interview guide as it evolved over three stages of interviews

Stage 1 (June 2021)	Stage 2 (December 2021)	Stage 3 (June 2022)
Firstly, can you tell me a bit about your practice and community?	Thanks for talking with me again	Thanks for talking with me again
Can you tell me a bit about your experiences working in primary care during the Covid‐19 pandemic? What have been some of the barriers and enablers?	How have things been progressing since we last spoke? Just to recall, we were in the state‐wide lockdown at that time. What is it like now? What are some of the current barriers and enablers?	How have things been progressing since we last spoke? Just to recall, the state‐wide lockdown had eased but we were still waiting on vaccinations. What is it like now? What are some of the current barriers and enablers?
Are you using any new models (in‐practice, on‐call roster, aged care and/or other specialist clinics)?	Have you noticed any changes to your practice, new models, networks?	Have you noticed any changes to your practice, new models, networks?
Have you changed anything about the way you are working with others in the community?	How are they working at the moment in terms of meeting the needs of the practice and the patients?	How are they working at the moment in terms of meeting the needs of the practice and the patients?
Were there any enablers or barriers to these models/networks or approaches in terms of meeting the needs of the practice and the patients?		
How are you managing vulnerable patient groups? Is there anyone you sense who needs more care or who is missing out?	Are there currently any groups who you think is presenting more or who might be missing out on care at the moment? What are the barriers and enablers to their care?	Are there currently any groups who you think is presenting more or who might be missing out on care at the moment? What are the barriers and enablers to their care?
Going forward, are you or the practice planning to integrate these models or approaches into your practice? If so, why? If not, why not? What are the barriers and enablers?	Going forward, are you or the practice planning to integrate these models, networks or approaches into your practice? If so, why? If not, why not? What are the barriers and enablers to their care?	Going forward, are you or the practice planning to integrate these models, networks or approaches into your practice? If so, why? If not, why not? What are the barriers and enablers to their care?
Tell me about how sustainable your workforce is and what training or other strategies you are using and why?	Tell me about how sustainable your workforce is and what training or other strategies you are using and why?	Tell me about how sustainable your workforce is and what training or other strategies you are using and why?
Can you tell me about your pandemic planning and what is working well or not and why?	Can you tell me about your pandemic planning? Have you used this time to review your planning and what have your learnt?	Can you tell me about your pandemic planning and what is working well or not and why?
How would you measure the success of your pandemic response?	Is there anything else you would like to add?	Is there anything else you would like to add?
Is there anything else you would like to add?		

### 2.3 Analyses

The analyses were informed by cross‐situational learning where learning about phenomenon occurs through multiple exposures where there may be exposure‐by‐exposure uncertainty and a lack of referential material (new pandemic conditions that are constantly changing) but meaning is sourced from combining multiple reference points.^26^ This theory, although currently rooted in word leaning, allows for describing learning in complex dynamic systems where practices were responding to unpredictable pandemic policies and community needs over time. Other specific health services theory which informed the analysis was diffusion of innovation where linkages between resource and knowledge systems related to the piloting of innovations relative to system readiness, practical experience of those innovations and the consequences of not acting.^27^ Further, the researcher interpreted the emergent findings using thematic analysis based on a deductive coding framework concerning access, challenges and innovations by time period. These were defined based on internationally acceptable frameworks^28^, ^29^ where these were available and further informed from the perspective of the research team and Medical Advisors within the PHN, to ensure they aligned with the context under study (Table 3). The framework used to define access is known to constitute supply (practice and service factors) and demand side (Patient) considerations.^28^

**TABLE 3:** Description of themes and sub‐themes

Theme	Description
Access	Matters related to patients getting clinical services that are needed consisting of practice/service side and patient side components^28^
Challenges	Issues that were difficult or new to deal with
Innovations	New ways of working that could be product, process, organisation or service^29^

To support a deeper understanding of the data, interviews were transcribed verbatim, read through repeatedly by the researcher and coded deductively.^30^ Interviews were analysed by stage first, and then as a longitudinal dataset to ensure that different challenges and innovations of the pandemic were reflected upon at each stage, and then considered longitudinally. Additions and alterations to the codes were made as blocks of 3‐4 transcripts were complete. As coding continued, extra codes were added, as deemed relevant.^31^

Emerging themes were discussed with Murray PHN staff in bi‐monthly meetings which were recorded in working notes and used to clarify, annotate, and organise material in a way that made sense, layering and reorganising themes, and drawing on the deep knowledge of Murray PHN staff.^31^ The Murray PHN staff regularly challenged the researcher's ideas, which helped to reduce subjective biases and test any assumptions.^32^ This aided validation. By discussing the themes regularly, issues were raised that led to deeper analysis, allowing for internal confirmation or disconfirmation. This process enabled thick description and triangulation until a final set of themes was agreed upon.

To aid interpretation of the data, a subtext of E, M or L was applied to define early (including registrar), mid or late career participants or P, Practice Managers. In the sub‐text, the practice staffing was reflected in full time equivalent and the rurality of the practice location as Reg (Modified Monash Model [MMM] 2–3 of 15–50,000 population and a regional centre^33^) or Rur (MMM 4–5, of <15,000 population^33^).

## 3 RESULTS

Of 12 practices contacted, 11 key informants from different practice and subregions enroled in the study and participated in at least two rounds of interviews (eight participants completed all three interview rounds [around 30 h of interview data]). Three participants were from solo community practices. Participants included a mix of female and male, mid‐, and late career GPs/Practice Managers and those in varying practice employment and practice sizes, including servicing aged care, hospitals, and multiple practices and working in different levels of remoteness (Table 4).

**TABLE 4:** Characteristics of participants and their practices^a^

Factor	Number
Sex	
Female	5
Male	6
Rurality (MMM)^33^	
MMM 2–3	4
MMM 4–5	6
Only practice in town	
Yes	3
No	7
Employment	
Principal or associate	3
Salaried or contractor	4
Registrar	2
Practice manager	2
Approx. practice size (adding all the FTE)	
4 FTE or less	4
5+ FTE	7
Service an on‐call roster, aged care and/or other specialist clinics in the town	
Yes	7
No (includes practice managers)	4

Abbreviations: FTE, full time equivalent; MMM, Modified Monash Model; PHN, Primary Health Network.

^a^ The practices that were invited spanned all rural regions of the large Murray PHN catchment.

The results are summarised in Table 5 and described below.

**TABLE 5:** Summary of results

Pandemic period	Challenges	Innovations
Stage 1	Absorbing new policies Designing care models that reduce health workforce exposure Limited personal protective equipment Time for planning Keeping services up to patients at risk New telehealth markets threatening fragmented care Planning telehealth services Less workforce due to their exposure/infection Reduce allied services for referral Different patient transport corridors	Driveway consultations Integration of blended models of care Innovative models for at‐risk cohorts to still see doctor FTF within guidelines Hub and spoke models where doctors use iPad and work with onsite nurse seeing patients, who then outreaches to doctor's home for paperwork sign off
Stage 2	Caring for increased population fleeing cities Caring for more complex patients with significant issues People attending with longer lists of problems Longer waiting lists requiring triage systems to ensure those most in need get care	Telehealth expansion and testing the application of phone and video‐modalities relative to context of practice, patient, and problem Business growth and patient centredness of phone consultations to manage non‐serious issues in rural areas where Internet coverage would be limited, and people are self‐employed
Stage 3	Planning the vaccination programme and managing cancellations and community hesitancy around vaccination Staff exhaustion and an ongoing unrelenting workload Chronic disease and mental health presentations more serious and wider cohorts impacted Social isolation and related impact of this for ageing people	Telehealth systems more embedded in practice, including to manage waiting lists and expand business opportunity Clarity about where and what telehealth is providing access, quality and efficiency

Abbreviation: FTF, face to face.

### 3.1 Stage 1—Challenges

In the first stage, when lockdowns were imposed and practices had to pivot to the use of telehealth (Table 1), some of the challenges to access involved practices spending long hours (by administrative and clinical staff) absorbing new policies and managing new roles and responsibilities around ways of delivering care safely during the pandemic, ‘*At the very early stage, there was a lot of anxiety and included in swabbing patients, and practice nurses were also worried. So we held lots of clinical meetings. We needed lots of planning*’. (P_6FTE_Reg). A shortage of personal protective equipment (PPE) placed the onus on practices to shield themselves from infection through removing vulnerable GPs from face‐to‐face services, screening and excluding potentially infectious patients and triaging the PPE that was available, ‘*Our main priority has been to exclude potentially affected people until they are tested or using telehealth, to minimise reliance on PPE*’ (L_14FTE_Reg). Challenges included communicating the latest advice and extending care to rural patients who relied on the local practice for such information. Meanwhile telehealth opened up a new market for healthcare and practices perceived they needed to maintain patient's connection to the local practice, ‘*Patients were wondering who was going to offer telephone consultations and who was going to do that well, so patients were looking around for services. There was a new market*’ (E_4FTE_Reg). Whilst new reimbursement was introduced for telehealth, it did not cover the needs for all patient cohorts and the full reliance on telehealth at this stage meant some patients were missing out, ‘*we couldn't do the long assessment, until these items became available by phone, we started using them*’ (E_4FTE_Reg). Some practices also experienced around 60% of their staff taking leave to get tested for Covid‐19. This reduced the doctors available to physically see patients, ‘*In last 9 weeks 60% of workforce have taken leave for swabs or go into isolation, because they have a cold, we are merely following guidelines*’. (P_4FTE_Reg) Doctors also had to navigate less access to allied medical services due to reduced staffing from provider vulnerability to Covid‐19, ‘*So our pathology service, we used to have 5 days they got cut back to 3 days, because the pathology service lost staff…*’ (L_6FTE_Rur) Traditional health referral pathways which are a critical part of accessing coordinated rural healthcare were also disrupted because of state‐imposed transfer rules, ‘*Within different regions under the ongoing state‐wide management, the patient pathway is really different for patient transfers*’ (L_8FTE_Rur).

### 3.2 Stage 1—Innovations

In the first stage, most of the innovations in practices related to infectious diseases prevention and control measures which were retrofitted each practice's infrastructure, skills, and interests. Some novel models emerged including driveway consults and carpark examinations, ‘*… we negotiated access to a driveway of a neighbouring property from which we could do a drive‐through clinic*’ (L_8FTE_Rur). Practices rapidly adopted telehealth care but showed the capacity to pivot to blended models based on using available space and systems in the practice to support safe face‐to‐face care for specific patient cohorts, ‘*For a proportion of around 15% people with chronic disease and mental health, a key part of our practice, during Covid‐19 we have been bringing complex patients into the practice, in a separate room and consulting online with the doctor online*’ (P_4FTE_Reg). One practice adopted a nurse‐led face‐to‐face hub and spoke care model (nurse based in the practice) supported by a doctor at home working online, ‘*patients went to the surgery and they linked to an iPad in my home. And we examined people via the nurse…In the vast majority of consultations, it didn't require us to be there*’ (L_5FTE_Reg). Within this model, the nurses managed service paperwork by taking this to the doctors at their respective homes at the end of the day for sign off.

### 3.3 Stage 2—Challenges

In the second stage, borders had re‐opened and the state‐wide lock‐down had eased (Table 1), access challenges continued to morph. At this stage some rural practices reported being stretched by the challenge of providing care to new rural residents who had moved from metropolitan locations due to the capacity to work from home, ‘*we have had a swell in our population from people who moved to [town] to get away from Melbourne. Their jobs went online, we picked up their medical care. So, we had increased workload*’ (L_8FTE_Rur). Otherwise, doctors and practice managers almost unanimously reported seeing more complex and mental health issues in patients at this time point, ‘*They are co‐morbid, like intense cases*’ (P_4FTE_Reg) and there were more significant presentations ‘*You don't see many people who don't have a real problem*’ (E_5FTE_Rur). Some noted these also related to the socio‐economic‐political climate related to the pandemic, ‘*The complex mental health appointments is soaring…Some are related to small business owners and young people are affected, some who came home from Melbourne [the city] to see out the lockdown. A lot have significant issues related to what's going on*’ (L_6FTE_Rur). The perception of delayed care during phase 1, and the perceived difficulty of getting access to medical appointments in stage 2, was viewed as people attending the practice with a longer list of things they needed addressed, resulting in long appointments which reduced available appointments, ‘…*most people are also coming with a list because it is taking longer to get an appointment*’ (M_6FTE_Rur). Another noted, ‘*Our appointment times have blown out up to several weeks for people to see their normal doctor*’ (L_6FTE_Rur).

### 3.4 Stage 2—Innovations

At the second stage, the innovations were driven by the expansion of telehealth reimbursements from the Australian government and the flexibility for practices to bill for it as they chose. Practices contemplated how to use the right mix of care modalities to support access. Many contemplated this innovation around patient limitations, patient characteristics, their healthcare needs and the doctor's satisfaction with the quality of care that could be provided, ‘*I like video…works well, once they have the hang of it, it is great especially as they live further away. But enabling people without the skills and equipment is the hard part. Even people with smart phones can't always use them for a consult, they also have trouble with the dexterity involved if they have issues with their hands*’. (L_6FTE_Rur). Reliability and efficiency were also factors those doctors considered, ‘*The consultations would be heaps better with video. But… The connection doesn't work, nurses don't know how to turn it on, the it is always “the dog ate my homework” … the reality is that the phone is so much more accessible*’. (L_8FTE_Rur). Doctors also perceived that telephone consultations allowed rural patients, including those with poor Internet connection, to access appointments when busy in self‐employed businesses or doing other activities, ‘*I have done telehealth while people are shopping, at work, on a tractor. In those places, they don't have access to Internet. It is efficient with the phone where we work*’ (L_8FTE_Rur).

### 3.5 Stage 3—Challenges

In the third stage, the national vaccination programme had started rolling out (Table 1). Given the government guidelines about the safety of vaccines changed over time, access challenges involved practice resources being deployed to provide information and support for anxious patients, ‘*first announcement about 50 years and below and Astra Zeneca, we have had cancellations and we had to manage this and the workforce… we are still seeing hesitancy*’ (P_6FTE_Reg). One respondent noted that vaccination was discussed in nearly every consultation ‘*In every consultation at the moment, we are talking about Astra Zeneca, and every consultation, everyone wants to discuss their risk for Covid‐19 vaccinations*’ (E_4FTE_Reg). Some commented they were ‘*Very tired, exhausted*—*the changes have been constant*’. (L_8FTE_Rur) Otherwise, access challenges included pressure on chronic diseases and mental health care since regular reviews had been delayed and the pandemic had introduced new life stressors, ‘*A lot of mental health stuff was exacerbated, and new mental health conditions emerged which was significant but the same old stuff otherwise*’ (E_5FTE_Rur). Others commented that these conditions seemed to express in a wider cohort, including the elderly and young people, ‘*There is more mental health coming up… It is not in any particular groups… I would say that it has been multi‐generational. There might be less social buffering and less interaction, and it affects people*’ (M_4FTE_Rur). Another respondent noted that the working age population was also impacted by Covid‐19 related stressors including, ‘*the impact on employment*’ and people getting ‘*burnt out*’ including from ‘*grants* [funding given to increase business] *in hospitality*’ and ‘*the huge pressure to work more*’ (L_6FTE_Rur). Another key issue was ageing people who had experienced more social isolation as a result of not seeing as many family or friends (border closures and people's reluctance to travel in the event of being made to spend up to 14 days in hotel quarantine) and now needed to access primary care to organise community supports to live independently, ‘*… we need to do heath assessments and encourage some referrals to get more home help, and they may have had family that was some distance away and now they are not visiting as much, so the changes have meant they need more help*’ (P_6FTE_Reg). One GP summarised conditions in the practice as being ‘*ridiculously busy*’.

### 3.6 Stage 3—Innovations

In the third stage, with more security around ongoing telehealth funding for telephone only, the innovations involved practices embedding telephone use into the practice model of mostly face‐to‐face services. This varied by doctor and practice depending on the sorts of patients the doctor/practice saw and additional areas of care they provided, ‘*Each doctor is different so they use it in different strategic ways… those doing mental health are wanting to use it differently to those doing more procedural work*’ (L_8FTE_Rur). It was also considered to depend on patient request relative to the doctor's assessment of need, ‘*They [doctors] do it depending on what the request is*’ (P_4FTE_Reg). Practices viewed telephone consultations as a useful adjunct service to support scripts, referrals, reviews and team meetings, ‘*…for chronic reviews. Where the patient is isolated, or repeat scripts or for following up results rather than getting someone in to discuss their issue, and you can organise the physio without them having to come in…*’ (L_8FTE_Rur). Another commented that they could be applied to help to manage waiting list ‘*…it helps us do the quick appointments and referrals and then we can manage waiting lists*’. (L_6FTE_Rur). Further, there was an example of a practice using telephone consultations to extend business opening hours as a means for patients to access more services, ‘*We are using the telehealth from 8–8:30 AM and then 5–5:30 when there is only a front of desk nurse, no screening staff at the door…working to increase revenue*’ (P_4FTE_Reg).

## 4 DISCUSSION

This longitudinal qualitative research provides some useful insights into the evolving challenges and innovations related to promoting access by rural General Practice during an intense year of policy change during the Covid‐19 pandemic. There are signs of major practice challenges and adaptations over time in relation to access, including managing major stressors such as maintaining community confidence and connection to local services, supporting the evolving needs of different patient cohorts and responding to local resource challenges (time spent planning, organising staffing and infrastructure and ensuring safety under pandemic conditions). In terms of theory about the diffusion of innovation, whilst the rural and regional General Practices showed that they have the capacity to mount a substantial response within pandemics, the number and size of changes at the macro level of the health system, such as new pandemic policies, can quickly overwhelm their systems.^27^ Further, their capacity to pursue innovations whilst managing challenges to service access, relies on state and national resource systems which may lag in their delivery. Therefore, rural and regional practices may have additional pressures to mount workarounds. Further, if pandemics emerge rapidly, practices are required to respond within the limits of their local resources, with some starting new models like driveway consultations, but these may not be universally possible depending on the community. Overall, with regard to rural and regional practice, they may have more challenges mobilising pandemic resources because they start pandemics with a greater relative service demand^3^ and the need to adjust pandemic responses to cope with a higher risk clientele who needs to travel further to get health services.^10^ In the Australian context, the PHNs and other stakeholders are likely to play a part in forecasting, advocating, and coordinating resource needs during pandemics, particularly for the rural and regional primary care sector. As a broker between the individual practices and the levels of government, PHNs have the potential to promote more of the intended outcomes of government pandemic policy and reduce more of the unintended consequences, also aggregating the government communications about the rapidly emerging pandemic policy settings to ensure that they make sense in the local context relative to resources.

Meanwhile, aligned with diffusion of innovation theory, the practices showed a strong imperative to evolve their service models to minimise consequences for the community and maximise business viability.^27^ This was most demonstrated through the application of telehealth over time, where it was tested and refined until it became more embedded in the practice with a clear purpose and to drive business revenue. Due to the wide variability in General Practices and communities and variation in issues like distance and costs, the rate and level of innovations at any one practice naturally varied greatly over the course of the study period. However, there was a universality in the voice of respondents, about their commitment to respond to local access needs likely due to the consequences of not doing so (Covid‐19 related morbidity and mortality). Consequences are a key driver of adopting innovation.^27^ With regard to telehealth, the macro systems change like the government's new opportunities for bill for telehealth appointments (initially requiring telehealth only models due to infection risks), met with significant effort by practices to trial and reflect on the use of telehealth in different forms in their local context, and then embed mostly telephone consultations for specific situations. The situations where it was deemed useful were mostly as telephone appointments within a hybrid service model, to manage waiting lists and promote convenience for rural patients, and to extend the business hours. This response to telehealth is strongly reflected through cross‐situational learning theory where emerging conditions were unknown and flexible response was needed to maintain services and adjust to emerging needs.^26^

The rapid policy changes and the size of the responsibility on small staffing within rural practices showed signs of producing a major workforce burden over the study period. This is a major concern in an environment where recruitment and retention challenges are already common.^34^ Rural workforce capacity should be a major consideration in ongoing pandemic planning for its major potential to impact accessible rural primary care. The findings suggest that policies around standing staff down who have been potentially exposed can have a major impact on rural access. Further, the major administrative and clinical planning capacity required for rural practices to respond to pandemic‐related care for a wide geographical population with high care needs, could be a major barrier to rural primary care sustainability. This reinforces the findings of a perspective about rural pandemic preparedness.^10^

The study also identified major changes in the needs of rural patients which could impact access to rural primary care. Over the study period, there were signs that rural population size and mobility patterns had the potential to change in line with other perspectives.^10^ Further, practices identified that there was an increasing demand for care to support community anxiety, manage more complex and mental health conditions, as well as demand by older frail patients due to physical and cognitive decline during periods of isolation. The study respondents noted the need to ensure access for patients with longstanding issues which were exacerbated by deferred reviews as well as handling additional caseload related to causes in the pandemic environment. Longer appointments were needed but once again there were not necessarily more doctors, and doctors tended to a higher proportion of more serious patients which could be a burden for sustaining their own well‐being (minimal simple caseload).

This study has limitations. It is exploratory only, based on a small sample of practices, including a mix of GPs and practice managers in different rural towns. Although the study explored a range of issues over time, due to the breadth of issues and variability of context, caution should be used when generalising these findings to other settings. Given the research showed that innovations emerged over time, it is recommended that follow‐up research is pursued.

## 5 CONCLUSION

This longitudinal qualitative research provides some useful insights into the evolving challenges and innovations related to promoting access by rural and regional General Practices during an intense year of policy change during the Covid‐19 pandemic. A common theme concerned reflexive action, to the extent to which practices had been on the journey to embed pandemic planning processes, digital health options and innovate to protect and support patients and themselves to sustain access. The research reinforces the need for resource coordination, support for communications, and forecasting and managing rural and regional primary care needs with levels of government, to achieve intended outcomes related to service access.

## CONFLICT OF INTEREST

The author was contracted and paid by Murray PHN. The author had full autonomy over the data collected and its interpretation.

## ETHICS STATEMENT

The study had ethics approval from Monash University, Project ID: 24918.

## Data Availability

Data are available by contacting the main author and subject to ethical approval.
